# Thymic gene expression analysis reveals a potential link between HIF-1A and Th17/Treg imbalance in thymoma associated myasthenia gravis

**DOI:** 10.1186/s12974-024-03095-7

**Published:** 2024-05-11

**Authors:** İlayda Altınönder, Mustafa Kaya, Sibel P. Yentür, Arman Çakar, Hacer Durmuş, Gülçin Yegen, Berker Özkan, Yeşim Parman, Amr H. Sawalha, Guher Saruhan-Direskeneli

**Affiliations:** 1 Department of Physiology, Istanbul Medical Faculty, Istanbul University, Istanbul, 34093 Turkey; 2 Department of Neurology, Istanbul Medical Faculty, Istanbul University, Istanbul, 34093 Turkey; 3 Department of Thoracic Surgery, Istanbul Medical Faculty, Istanbul University, Istanbul, 34093 Turkey; 4 Department of Pathology, Istanbul Medical Faculty, Istanbul University, Istanbul, 34093 Turkey; 5 Division of Rheumatology, Department of Pediatrics, University of Pittsburgh, Pittsburgh, PA USA

**Keywords:** Thymoma, Thymus, Myasthenia gravis, Hypoxia-inducible factor-1

## Abstract

Myasthenia gravis (MG) is an immune-mediated disease frequently associated with thymic changes. Increased T helper 17 (Th17) cell activity and dysfunctional regulatory T (Treg) cells have been demonstrated in subgroups of MG. On the other hand, hypoxia-inducible factor 1 (HIF-1) has been shown to regulate the Th17/Treg balance by inducing Th17 differentiation while attenuating Treg development. To identify the underlying mechanisms of different thymic pathologies in MG development, we evaluated thymic samples from thymoma-associated myasthenia gravis (TAMG), MG with hyperplasia (TFH-MG) and thymoma without MG (TOMA) patients. Differential gene expression analysis revealed that TAMG and TFH-MG cells are associated with different functional pathways. A higher *RORC/FOXP3* ratio provided evidence for Th17/Treg imbalance in TAMG potentially related to increased *HIF1A*. The hypoxic microenvironment in thymoma may be a driver of TAMG by increasing *HIF1A*. These findings may lead to new therapeutic approaches targeting *HIF1A* in the development of TAMG.

**Supplementary Information:**

The online version contains supplementary material available at 10.1186/s12974-024-03095-7.

## Introduction

Myasthenia gravis (MG) is an immune-mediated neuromuscular disease frequently associated with thymic changes and autoantibodies against the acetylcholine receptor (AChR). While the most common concomitant thymic pathology with MG is thymic follicular hyperplasia (TFH), a smaller subgroup (10–20%) of MG patients presents with thymoma, which is clinically indistinguishable from TFH-MG.

Thymoma is a rare epithelial tumor of the thymus. Thymoma patients frequently co-exhibit autoimmune disorders, of which thymoma-associated myasthenia gravis (TAMG) is the most common, occurring in 64% of all thymoma patients [1]. Thymomas are classified into several subtypes according to the morphology of the tumor cells and the proportion of associated immature T cells (i.e., WHO type A, AB, B1, B2, B3, and other rare subtypes) [2]. TAMG is more common with type B than type A or AB thymomas [2–5] and is almost always accompanied by anti-AChR antibodies, as also frequently observed in other forms of MG with TFH.

Tolerance breakdown by intrathymic mechanisms, including abnormal T cell selection and activation, has been intensively investigated in MG [6, 7]. Defective tolerogenic features (e.g., the lack of AIRE expression) [5, 8–10] and reduced intratumoral generation of regulatory T (Treg) cells [11, 12] have been considered possible mechanisms for MG development. The analysis of T cell subset composition in the blood revealed an increased proportion of mature CD4 + and CD8 + T cells in TFH-MG and TAMG compared to healthy controls [13, 14]. Similar results were also reported in thymic samples, showing that the presence of TFH-MG or TAMG cells was significantly linked to high levels of mature naive CD4 + T cells [15]. Furthermore, an export of increased autoreactive CD4 + T cells, accompanied by reduced numbers of Treg cells, was demonstrated in TAMG in comparison to TFH-MG and thymoma patients [12]. The functional and effective balance between Treg cells and effector T cells is lost in the thymus of anti-AChR + TFH-MG patients as well [16]. However, there was no significant difference between these groups in the periphery [11, 17, 18]. Finally, an increase in IL-17-related activity and a decrease in Treg cell development have also been demonstrated in TAMG or thymoma without MG (TOMA) [19, 20]. All these data suggest possible Treg- and IL-17-related imbalance and potential activation of Th17 cells in the thymus of MG patients [21]. The increase in active IL-17-expressing cells in the MG thymus may be a consequence of the impaired ability of Treg cells to suppress effector T cells. Therefore, “plastic” Treg cells in AChR + MG may become Th17-like ex-Treg cells and contribute to the rise in the IL-17 concentration in the thymus [22].

Solid tumors commonly have a hypoxic microenvironment because of rapid proliferation. To adapt to low O_2_ levels, cells activate several survival pathways, such as the hypoxia-inducible factor-1 (HIF-1) signaling pathway. HIF-1, as a transcription factor, activates over 100 downstream genes that regulate cell survival, proliferation, metabolism, and angiogenesis. Its *α*-subunit (HIF-1*α*) is hydroxylated by prolyl hydroxylase enzymes (PHDs, also known as *EGLN*s) and degraded by the von Hippel − Lindau protein (pVHL) complex under normal O_2_ tension. However, under hypoxic conditions, PHD3 activity is reduced, leading to HIF-1*α* stabilization and HIF-1 activation [23]. Importantly, HIF-1 has been shown to modulate Th17/Treg balance by favoring Th17 development while inhibiting Treg differentiation. HIF-1 transcriptionally activates RORγt, a transcription factor that plays a central role in Th17 differentiation and induces IL-17 A by cooperating with RORγt and p300. Moreover, HIF-1 targets FOXP3, the specific transcription factor of Treg cells, for ubiquitination and proteasomal degradation [24].

We hypothesized that the hypoxic tumor microenvironment of thymoma and the subsequent changes in HIF-1-related mechanisms could have disease-causing or disease-modulating effects on the development of TAMG in thymoma patients. In this study, we assessed gene expression in thymic tissues from TAMG and TFH-MG patients, as well as TOMA patients, with two different approaches. Global expression differences and targeted expression analysis were performed in thymic tissue samples to confirm disease-related/promoting changes in MG. We report that HIF-1 might play an important role in the development of TAMG but not TFH-MG.

## Materials and methods

### Patients and controls

In this study, thymic tissues from patients undergoing thymectomy at the Istanbul Medical Faculty Hospital (Istanbul, Turkey) between 2015 and 2021 were used. The diagnosis of MG was based on clinical presentation and electrophysiological examination.

Thymic samples from thymectomized MG patients with pathologically proven thymoma (TAMG, *n* = 7, median age: 33, 16–47 years old, 4 women and 3 men) or follicular hyperplasia (TFH-MG, *n* = 8, median age: 24, 23–66 years old, all women) were included in the sequencing experiments. TFH-MG patients were younger than TAMG patients. All patients had AChR antibodies, and half of the patients had immunosuppressive (IS) treatment before thymectomy in both groups (3 of 7 thymoma vs. 4 of 8 hyperplasia patients).

The WHO classification was used for the thymomas [25]. Only one patient with type B1 thymoma and 6 patients with B2 thymoma were included in the thymoma tissues (Table 1).

**Table 1:** Demographic information of the tissue samples (15) included in the sequencing experiments

DNA (ID)	Age at TX	Sex	Anti-AChR titer (nmol/liter)	Immunosuppressive	Group	Pathological classification (WHO)
Tissue samples						
10,970	32	F	(+)	no	TAMG	B2
10,971	23	M	12.4	no	TAMG	B2
10,685	66	M	4.2	no	TAMG	B1
10,866	42	F	(+)	no	TAMG	B2
10,807	28	F	130	yes	TAMG	B2
12,175	44	F	14.3	yes	TAMG	B2
12,183	33	M	18	yes	TAMG	B2
10,814	27	F	84.4	no	TFH-MG	
10,850	16	F	19.3	no	TFH-MG	
12,074	22	F	7.1	no	TFH-MG	
12,333	17	F	9.8	no	TFH-MG	
10,827	22	F	11.7	yes	TFH-MG	
12,076	26	F	8.8	yes	TFH-MG	
12,287	47	F	(+)	yes	TFH-MG	
10,809	40	F	12.8	yes	TFH-MG	

TX: thymectomy

In the targeted expression analysis group, 38 thymic tissue samples from 12 TAMG, 14 TFH-MG and 12 thymoma without MG (TOMA) patients were included (Table 2). All MG patients had AChR autoantibodies and had not received IS treatment at the time of thymectomy. Among the patient groups, TFH-MG patients were significantly younger than TAMG and TOMA patients (*p* < 0.001). The proportion of women was slightly higher in the TFH-MG subgroup (86 vs. 50 and 67%). Written informed consent was obtained from all participants. This study was approved by the Institutional Ethical Committee of Istanbul Medical Faculty and performed in accordance with the latest version of the Helsinki Declaration.

**Table 2:** Demographic information of the tissue samples included in the qPCR experiments

DNA (ID)	Patients	Age of thymectomy	Sex	WHO histological thymoma classification
10,250	TAMG 1	42	M	B2
10,254	TAMG 2	39	M	B2B3
10,685	TAMG 3	66	M	B1
10,866	TAMG 4	42	F	B2
10,971	TAMG 5	23	M	B2
12,282	TAMG 6	43	F	B2
12,653	TAMG 7	57	F	B1
12,676	TAMG 8	45	F	B2
12,710	TAMG 9	62	M	B2B1
12,711	TAMG 10	47	M	B2B3
10,970	TAMG 11	32	F	B2B3
12,701	TAMG 12	45	F	B2B1
Median age (range)		44 (23–66)		
10,237	TOMA 1	59	F	AB
10,617	TOMA 2	53	F	AB
10,810	TOMA 3	43	F	AB
10,933	TOMA 4	43	F	B2B3
10,948	TOMA 5	77	F	B1B2
12,098	TOMA 6	52	F	B2
12,239	TOMA 7	61	M	B2B3
12,274	TOMA 8	46	M	B2
12,704	TOMA 9	27	F	AB
10,953	TOMA 10	62	M	B1B2
12,334	TOMA 11	63	M	B2B3
12,344	TOMA 12	62	F	B1B2
Median age (range)		56 (27–77)		
10,814	TFH 1	27	F	
12,029	TFH 2	12	F	
12,048	TFH 3	25	F	
12,074	TFH 4	22	F	
12,214	TFH 5	25	F	
12,250	TFH 6	26	F	
12,251	TFH 7	31	F	
12,319	TFH 8	22	F	
12,328	TFH 9	32	F	
12,662	TFH 10	37	M	
12,709	TFH 11	26	M	
12,717	TFH 12	21	F	
10,962	TFH 13	42	F	
12,333	TFH 14	17	F	
Median age (range)		25,5 (12–42)		

### RNA library preparation and sequencing

RNA sequencing libraries were prepared using an Illumina TruSeq RNA Exome kit (Illumina, Inc., San Diego, CA, USA) according to the manufacturer`s protocol. The RNA concentration was measured with a Nanodrop 2000c spectrophotometer (Thermo Scientific Inc., Waltham, MA, USA). Integrity was assessed using an Agilent 2200 Tapestation instrument (Agilent Technologies, Santa Clara, CA, USA), and the percentages of fragments larger than 200 nucleotides (DV200) were calculated. RNA samples (20 ∼ 100 ng) were used as input based on the DV200 value. First strand cDNA syntheses were performed at 25 °C for 10 min, 42 °C for 15 min and 70 °C for 15 min using random hexamers and ProtoScript II Reverse Transcriptase (New England BioLabs Inc.). In second-strand cDNA synthesis, the RNA templates were removed, and a second replacement strand was generated by incorporating dUTP (in place of dTTP to keep strand information) to generate ds cDNA. The blunt-ended cDNA was cleaned from the second strand reaction mix with beads. The 3` ends of the cDNA were then adenylated, followed by the ligation of indexing adaptors. PCR (15 cycles of 98 °C for 10 s, 60 °C for 30 s and 72 °C for 30 s) was used to selectively enrich those DNA fragments that had adapter molecules on both ends and to amplify the amount of DNA in the library.

The library was qualified using an Agilent 2200 Tapestation instrument and quantified using a QuantiFluor dsDNA System (Promega). A 4-plex pool of libraries was made by combining 200 ng of each DNA library. The pooled DNA libraries were then mixed with capture probes to target regions of interest. Hybridization was performed by 18 cycles of 1-minute incubation, starting at 94 °C and then decreasing 2 °C per cycle. Then, streptavidin-coated magnetic beads were used to capture probes hybridized to the target regions. The enriched libraries were then eluted from the beads and prepared for a second round of hybridization and capture to ensure high specificity of the capture regions. The enriched libraries were amplified by a second 10 cycles of PCR amplification (98 °C for 10 s, 60 °C for 30 s and 72 °C for 30 s) followed by bead clean up. The final libraries were validated using Agilent High Sensitivity D1000 ScreenTape on an Agilent 2200 TapeStation instrument. The size distribution of the library ranged from approximately 200 bp–1 kb. Libraries were normalized, pooled and subjected to cluster and pair read sequencing for 150 cycles on a HiSeqX10 instrument (Illumina, Inc. San Diego, CA, USA) according to the manufacturer’s instructions.

### Data analysis

Data were analyzed with the Illumina BaseSpace app suite (https://www.illumina.com/products/by-type/informatics-products/basespace-sequence-hub/apps/rna-seq-alignment.html). The sequencing reads were aligned to Homo sapiens hg19 using STAR aligner. Salmon was used for quantification of reference genes and transcripts. Manta was used to detect gene fusions. GC and mean coverage information for every target was computed using Picard. The gene counts, gene FPKMs, principal component analysis and differential expression results were produced by DESeq2.

For gene expression, the data were analyzed using one-way ANOVA, and differentially expressed genes (DEGs) were defined as transcripts with more than 1.2-fold change (FC) of expression at a false discovery rate (FDR) adjusted P value less than 0.05.

Pathway analysis of DEGs was performed for gene enrichment analysis using the Ingenuity Pathway Analysis tool (IPA 8.0, Ingenuity Systems, and Redwood City, CA).

### RNA extraction and cDNA synthesis

Thymic tissues were preserved first in RNAlater® and then stored in liquid nitrogen. RNA was extracted from whole thymic tissues following the protocol recommended by the manufacturer (Zymo Research Direct-zol Miniprep Kit). The RNA pellet was dissolved in water and stored at – 80 °C until cDNA synthesis.

All RNA samples were quantified using a spectrophotometer (NanoDrop, ND-2000, Thermo Fisher Scientific). The ratio of absorbance at 260 nm and 280 nm was used to determine RNA purity. Samples with a ratio ranging between 1.7 and 2.1 were included in the relative quantitative PCR (qPCR) experiments.

cDNA was synthesized through a reverse transcription reaction according to the manufacturer’s protocol (Thermo Scientific) and stored at − 20 °C until qPCR.

### Quantitative PCR (qPCR)

qPCR was performed on a LightCycler 480 (Roche Laboratories) with SYBR green using 30 ng (3 µl) cDNA, 0.5 µl of 10 µM each primer and 6.5 µl Mastermix (SensiFAST™ SYBR® No-ROX Kit, Meridian Bioscience). Relative expression of selected genes was evaluated by comparing to 18 S and HPRT as house-keeping genes by real-time qPCR. The experimental run protocol was as follows: preincubation at 95 °C for 8 min; amplification for 45 cycles (15 s at 95 °C, then 5 s at 30 °C, final extension for 10 s at 72 °C); and final melting curve analysis. For each gene, the specificity of the PCR product was assessed by verifying a single peak in melting curve analysis.

The list of the genes and the primers (Sentromer DNA Technologies) used in the study is shown in Supplementary Table 1.

### Statistical analysis

Efficiencies for individual samples in qPCR were calculated using the LinRegPCR program [26]. Relative gene expression was calculated using E^−ΔCT^. Differences between two different groups were compared by unpaired (Mann‒Whitney *U* test) nonparametric tests. Values of *p* < 0.05 were considered significant. The results are presented as the mean ± SEM.

## Results

### Global sequencing of thymic tissues of TAMG and TFH-MG patients

Although the clinical picture of AChR + MG can be phenotypically similar in both TAMG and TFH-MG, thymic pathologies implicate differential effects of the thymus. To compare the possible effects of pathologies in these disease subgroups, tissue samples from 7 TAMG and 8 TFH-MG patients were evaluated in this study. The TFH-MG group consisted of women, whereas there was no significant dominance of women in the TAMG group. TAMG patients were 9 years older than TFH-MG patients. In total, 43% and 50% of patients were treated with IS agents in the TAMG and TFH-MG groups before thymectomy. As the proportions of patients on treatment were similar in the thymoma and follicular hyperplasia groups, the treatment effect was not evaluated separately in the groups.

RNA sequencing and comparison between expressed genes revealed 51 DEGs in the whole group. Among these genes, the expression of 24 was higher in TAMG, whereas 27 genes were upregulated in TFH-MG tissue samples (Supplementary Table 2).

To independently validate gene expression changes in the thymus, 3 DEGs with FC > 1.8 and detailed/functional annotation (*TF, GATA3, FCLR2* and *FLT4*) were selected for further validation by qPCR using RNA samples from 16 MG patients (8 TAMG and 8 TFH-MG). The expression levels of these four mRNAs in TAMG cells were not significantly different from those in TFH-MG cells, among which *TF, GATA3, FCLR2* and *FLT4* displayed 1.8-, 0.55-, 0.14-, and 1.89-fold differences in expression, respectively. Out of the 4 DEGs evaluated, only *FCLR2* was significantly different between cases and controls. However, the TFH-MG group had higher expression of *FCLR2* than the TAMG group (3.1 vs. 0.5, *p* = 0.015).

### Different pathways are associated with TAMG and TFH-MG cells

To better understand the role of differentially expressed genes in underlying mechanisms in TAMG, we performed pathway analyses for upregulated mRNAs with an absolute fold-change ≥ 2 and identified pathways known to be important in TAMG [27]. The analysis of these 51 genes showed high connectivity between transcripts with activation of immune function, inflammation, and enrichment of canonical pathways, such as TREM1 and Toll-like receptor signaling (Table 3A).

**Table 3A:** Pathways deteceted with differentially expressed genes

Name	p value	Overlap
Fc Receptor-mediated Phagocytosis in Macrophages and Monocytes	6.44E^− 04^	3.2% 3/94
IL-8 Signaling	5.54E^− 03^	1.5% 3/200
Pyridoxal 5’-phosphate Salvage Pathway	6.28E^− 03^	3.0% 2/66
Remodeling of Epithelial Adherence Junctions	6.66E^− 03^	2.9% 2/68
Macropinocytosis Signaling	8.25E^− 03^	2.6% 2/76

Gene enrichment analysis of these findings implicated *ESR1* (estrogen receptor alpha)-, *NFKB* (nuclear factor kappa B)-, *HNF4A* (hepatocyte nuclear factor 4 alpha)-, and *EGLN3* (PHD3, prolyl hydroxylase enzymes)-related activities as increased in TAMG patients compared to TFH-MG patients (Table 3B).

**Table 3B:** Some of the differentially expressed genes between TAMG and THP-MG samples and the related pathways derived on these genes

Genes	log2 (Fold Change)	Std. Err. Log2	q Value	Pathways			
TBC1D3D (TBC1 domain family member 3D)	25.45	2.98	0.000	GLN3 (PHD3, prolyl hydroxylase enzymes)			
TBC1D3I (TBC1 domain family member 3I)	25.45	2.98	0.000				
TBC1D3K (TBC1 domain family member 3 K)	25.45	2.98	0.000				
HSD17B3 (hydroxysteroid 17-beta dehydrogenase 3)	3.51	0.87	0.024	HNF4A (hepatocyte nuclear factor 4 alpha)			
LRRK2 (leucine rich repeat kinase 2)	1.36	0.36	0.044				
MRNIP (MRN complex interacting protein)	1.75	0.34	0.000				
ACCS (1-amino-cyclopropane-1-carboxylate synthase homolog)	1.19	0.32	0.049				
FAM153A (family with sequence similarity 153 member A)	2.77	0.68	0.022				
OSBPL10 (oxysterol binding protein like 10)	2.27	0.61	0.049	NFKB (nuclear factor kappa B)			
POU6F1 (POU class 6 homeobox 1)	2.15	0.51	0.015				
FLT4 (fms related receptor tyrosine kinase 4)	1.86	0.38	0.001				
PRKCE (protein kinase C epsilon)	1.43	0.36	0.029				
FCRL2 (Fc receptor like 2)	3.35	0.81	0.020	ESR1 (estrogen receptor alpha)			
SNORA71D (small nucleolar RNA, H/ACA box 71D)	5.84	1.51	0.036				
SNORA8 (small nucleolar RNA, H/ACA box 8)	3.65	0.97	0.046				
PPFIBP2 (PPFIA binding protein 2)	1.27	0.31	0.024				
PPIP5K1 (diphosphoinositol pentakisphosphate kinase 1)	1.2	0.3	0.022				

### Targeted gene analysis reveals increased *HIF1A* expression in TAMG

Increased Th17 and decreased Treg cell activity were demonstrated in TAMG [19, 20]. HIF-1 transcriptionally activates RORγt, favoring Th17 cells while inhibiting Treg cells by targeting FOXP3 for degradation [24]. PHD3 (*EGLN3*) is a prolyl hydroxylase enzyme that regulates HIF-1 levels by hydroxylating HIF-1α subunits when O_2_ is available [28]. Increases in PDH3 mRNA levels have been demonstrated in hypoxia [29]. Under hypoxic conditions, HIF-1*α* is stabilized and binds to hypoxia-responsive elements (HREs) in the promoters of its target genes. HIF-1 requires its coactivator p300 (encoded by *EP300*) to exert its transcriptional activity [30].

**Fig. 1:**
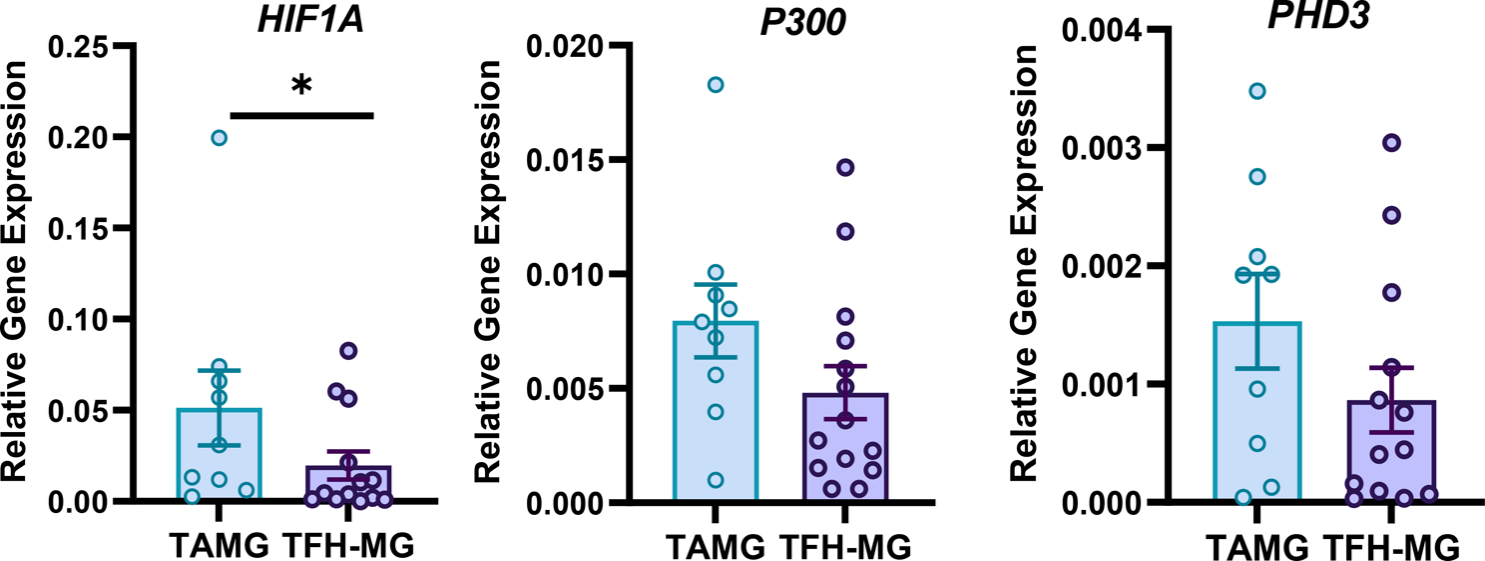
Relative expression of *HIF1A* (TAMG *n* = 9, TFH-MG *n* = 13) and related *EP300* (TAMG *n* = 9, TFH-MG *n* = 14) and *EGLN3* (TAMG *n* = 9, TFH-MG *n* = 13) genes in thymic samples of TAMG and TFH-MG patients. *p = 0.049

Increased *EGLN3* (PHD3) expression in the sequence analysis of TAMG samples supported the involvement of the HIF-1 signaling pathway in TAMG. To validate this finding, we measured *HIF1A, EGLN3* and *EP300* expression in thymic tissue samples from TAMG or TFH-MG patients by using qPCR. *HIF1A* expression was significantly increased (2.6-fold higher) in TAMG cells compared to that in TFH-MG cells (*p* = 0.049). Interestingly, the overexpression of *HIF1A* correlated with its downstream signaling pathway. The expression levels of *EP300* and *EGLN3* were both 1.9-fold higher in TAMG cells than in TFH-MG cells, without reaching significance (Fig. 1).

### The Th17 lineage is involved in TAMG

**Fig. 2:**
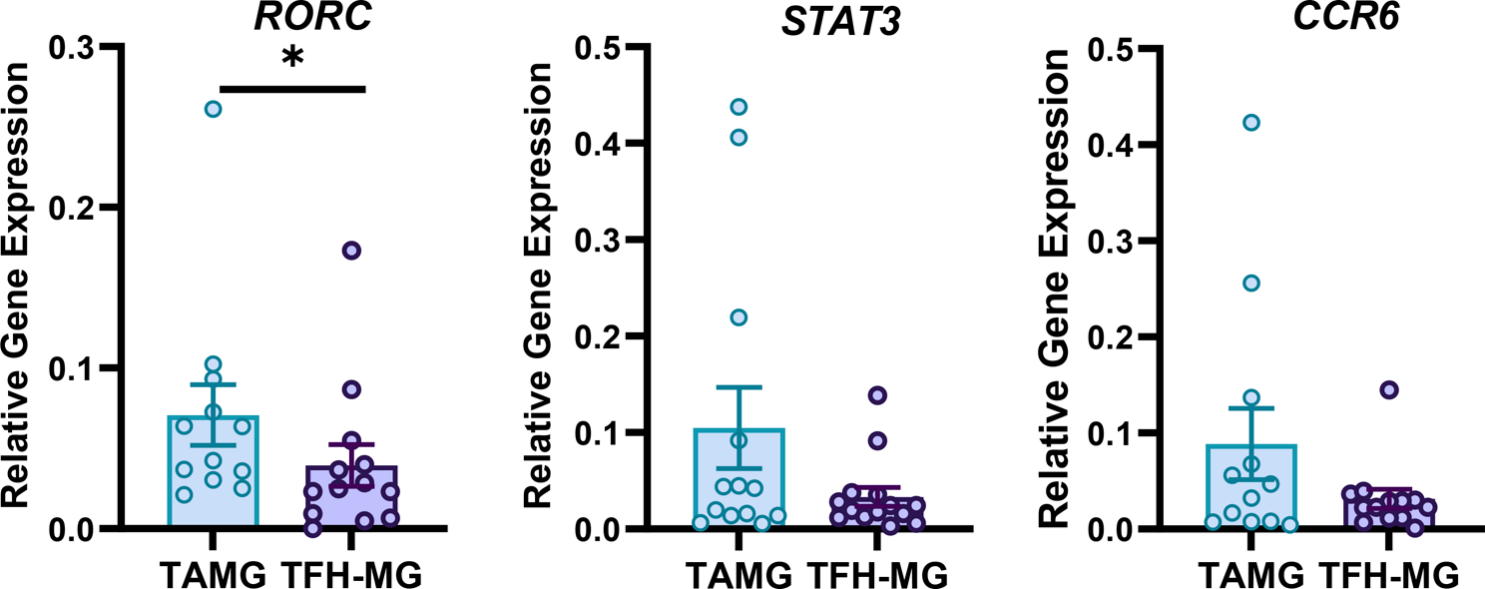
Relative expression of selected genes for IL-17-related activity: *RORC* (TAMG *n* = 12, TFH-MG *n* = 13), *STAT3* (TAMG *n* = 13, TFH-MG *n* = 14) and *CCR6* (TAMG *n* = 12, TFH-MG *n* = 13) expression in thymic samples of patients. *p = 0.039

The expression of Th17-related genes was analyzed to investigate whether elevated levels of HIF-1 may have led to increased Th17 involvement in TAMG. RORγt is a Th17 lineage-specific transcription factor and is encoded by the *RORC* gene. STAT3 is one of the transcription factors that regulates RORγt and IL17A [31], while CCR6 is a surrogate Th17 marker [32]. *RORC* expression was significantly increased in TAMG cells compared to that in TFH-MG cells (1.8-fold, *p* = 0.039). Moreover, genes performing as drivers of the Th17 response revealed a trend for an increase in expression levels. *STAT3* and *CCR6* expression in TAMG cells was 3.2-fold and 2.8-fold higher than that in TFH-MG cells (Fig. 2).

### Treg-related genes are underexpressed in TAMG

**Fig. 3:**
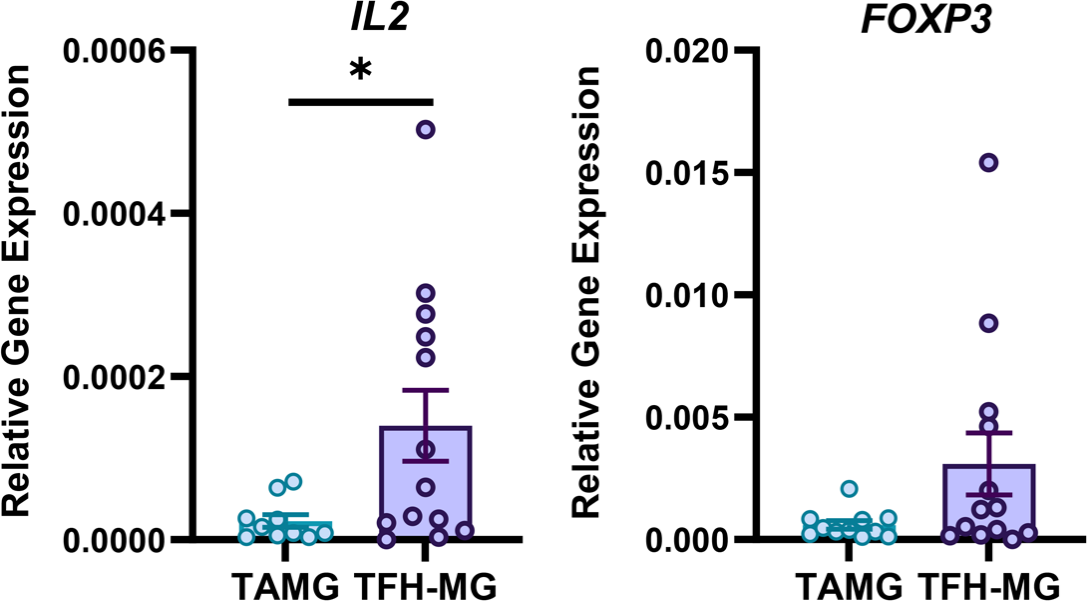
Relative expression of selected genes for Treg-related activity: *IL2* (TAMG *n* = 10, TFH-MG *n* = 13) and *FOXP3* (TAMG *n* = 11, TFH-MG *n* = 13) in thymic samples of patients. *p = 0.047

Since HIF-1 inhibits Treg differentiation [24], we performed a similar analysis for Treg-related genes. In the thymus, IL-2 is essential for Treg differentiation [33]. The expression of *IL2* was significantly decreased in TAMG cells compared to that in TFH-MG cells (0.16-fold, *p* = 0.047). Similarly, *FOXP3* expression in TAMG revealed a decreasing trend compared to that in TFH-MG (0.19-fold) (Fig. 3). The expression levels of Treg-related molecules, such as CTLA-4 and GITR, were similar in both groups (Supplementary Fig. 1).

### Impairment of the Th17/Treg balance in TAMG

**Fig. 4:**
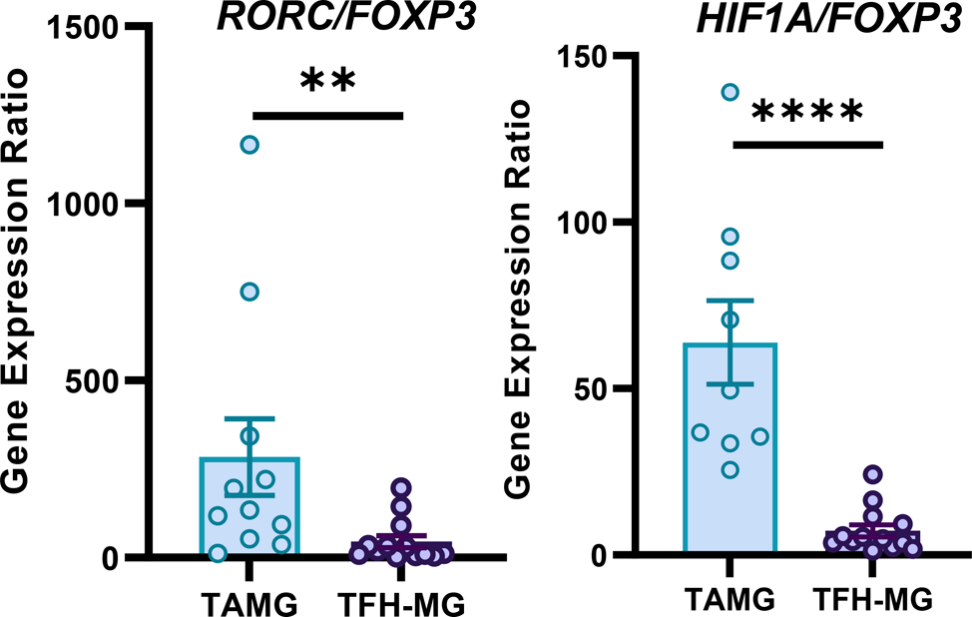
Relative expression of selected genes for Treg-related activity: *IL2* (TAMG *n* = 10, TFH-MG *n* = 13) and *FOXP3* (TAMG *n* = 11, TFH-MG *n* = 13) in thymic samples of patients. *p = 0.047

This expression analysis indicated a disturbance of the Th17/Treg balance in the pathogenesis of TAMG. To support this finding, we evaluated both changes in conjunction by analyzing the ratios of the relative expression of *RORC* to *FOXP3* and *HIF1A* to *FOXP3*. The ratios of both *RORC* and *HIF1A to FOXP3* were significantly higher in TAMG cells than in TFH-MG cells (*p* = 0.003 and *p* < 0.001, Fig. 4).

### No difference in expression levels between TAMG and TOMA

To rule out/dissect thymoma-related changes, we also compared the gene expression in a thymoma (TOMA) cohort with that in the TAMG samples. We did not observe any statistically significant differences in gene expression between TAMG and TOMA (Supplementary Fig. 2).

## Discussion

Thymoma is a rare epithelial tumor that originates from the thymus and is frequently associated with MG. The role of thymoma in the development of concomitant MG is not fully understood. As thymic follicular hyperplasia or even thymic age-dependent atrophy can also be observed alongside MG development, thymus pathologies may not be inducing but only accompanying the disease. In this study, the thymus was approached as the decisive organ for disease development, and thymic pathologies were compared with each other to determine possible effects on the disease. By RNA-sequencing analysis of thymic samples, several differentially expressed genes and pathways were detected, which were then partially confirmed by expression analysis. Thymoma-related changes have pointed to HIF-1 upregulation at the transcriptional level, which may be the driver of the previously described IL-17/Treg imbalance in the thymus.

Thymoma tissue has been investigated intensively for its role in autoimmunization against autoantigens in MG. Skeletal muscle antigens have been reported as potential autoantibody targets in TAMG [34], and incomplete autoantigens, proteins cross-reacting with AChR, titin, and ryanodine receptor epitopes have also been demonstrated in MG [10, 35–37]. A further comprehensive analysis of thymic epithelial tumors revealed that TAMG was associated with intratumoral overexpression of genes that show sequence similarity with major autoimmune targets as well [38]. Similar to our results, the expression of genes involved in immunity showed no significant differences between TAMG and TOMA in the TCGA thymoma study [38]. In a more recent report utilizing single-cell RNA-seq and immunohistological examination of MG-type thymoma specimens, neuromuscular expression was shown to be limited in a subpopulation of medullary thymic epithelial cells (mTECs). Therefore, TAMG develops in an atypical immune microenvironment that leads to B cell maturation and ectopic expression of neuromuscular antigens by epithelial cells [39]. Increased expression of neuromuscular antigens could not be demonstrated in the present study. The relatively low number of samples and heterogeneity of cellular components in whole thymic tissue may account for the discordance with the reported findings. Although not significant, gender and age differences among the study groups might have also influenced the different genetic expression patterns in the present study.

A previous analysis of functional pathways with metabolic and immunological relevance has also shown differences in TAMG compared to TOMA. The comparison of expression profiles of simple thymoma with TAMG identified candidate genes/pathways [27]. The link between the nuclear factor-κB/autoimmune regulator pathways and MG pathogenesis was supported in this study by gene enrichment analysis. Further analysis of thymic cell groups for their contribution to this pathway is needed.

Potentially relevant functional pathways were also compared among thymoma histological subtypes (type AB vs. B2 thymomas) in the TCGA data set, showing that different molecular pathways are upregulated in different thymoma histotypes [40]. In the present sample set, the group was relatively homogenous, and differences between thymic histotypic pathologies could not be evaluated.

Targeted expression analysis in this study revealed *HIF1A* upregulation in TAMG, potentially driving the previously described imbalance between Th17 cells and Tregs in the thymus. HIF-1 is a heterodimeric transcription factor consisting of a highly regulated oxygen-sensitive HIF-1α subunit (*HIF1A*) and a constitutively present β subunit (*HIF1B*) [41]. The hypoxic microenvironment and the distorted immune landscape of thymoma may be responsible for the failure of T cell tolerance and TAMG initiation. In a recent study, *HIF3A* was also differentially expressed in thymoma patients with MG [42], pointing to the relationship between hypoxia and TAMG. Increased expression of HSP60 demonstrated in thymoma tissues may also be involved in the response of the tumor tissue to hypoxic stress [10]. Indeed, *HIF1A* expression was also increased in TAMG cells compared to that in TFH-MG cells in the present study. As reported earlier as a risk factor [43], TAMG patients were older than TFH-MG patients in this cohort. In the TFH-MG group, only early onset MG patients were included, as the older patients are not be thymectomized. The observed difference may be due to the age difference between the groups, but the fact that this difference was not observed in the TOMA group emphasizes the role of the thymus in the development of MG in TAMG.

HIF-1-related mechanisms are regulated by hydroxylation with EGLN2 and EGLN3.

The VHL gene encodes pVHL, which is a part of the E3 ubiquitin ligase complex and controls the cellular levels of HIF-1α. Intracellular prolyl hydroxylases (PHD2, PHD3) hydroxylate HIF-1α under normoxic conditions. The VHL protein complex binds to the hydroxylated HIF subunits, causing the degradation of HIF-1α. Von Hippel‒Lindau (VHL) disease is a hereditary condition with a germline pathogenic variation of the VHL gene. Loss of function of the VHL complex in VHL disease causes HIF-1α accumulation and thus pseudohypoxia. Cases with both VHL disease and MG have been reported recently [44]. Although these cases might be coincidental, a link between VHL disease and MG with overlapping molecular mechanisms has also been proposed.

Another indirect finding in the same direction has been reported in TAMG. USP29, a deubiquitylase that stabilizes HIF-1 and posttranscriptionally regulates RORγt, was increased in TAMG patients [45]. Thus far, the associations of HIF-1-related mechanisms with the pathogenesis of TAMG need further clarification.

The present study has certain limitations. First, the number of samples included in the sequencing group was limited, and the proportion of patients receiving immunosuppressive (IS) treatment was relatively high. However, as the proportion of patients with treatment was similar in the TFH-MG and TAMG groups, treatment itself is unlikely to affect the results in the whole group. Second, the present findings are at the transcriptional level and need further verification at the protein level. Thirdly, the age-related changes in the thymic tissue could not be ruled out in the sample groups, as the ages of the patients were different in the study goups directly inherent to the disease subgroups.In conclusion, we suggest that the hypoxic environment of thymoma is a potential driver of TAMG but not TFH-MG. *HIF1A* upregulation in thymic tissues of TAMG patients correlated with a higher *RORC/FOXP3* ratio, indicating its role in Th17/Treg imbalance. Further studies focusing on the causality between HIF-1 and TAMG development are warranted. A better understanding of the link between hypoxic changes and the thymoma microenvironment could help in the development of novel treatment strategies for TAMG.

***This study was supported by TÜBİTAK (116S317) and I.U. BAP (36227*** ***and*** ***36288).***

### Electronic supplementary material

Below is the link to the electronic supplementary material.

Supplementary Material 1

## Data Availability

All datasets can be accessed by request from the authors.

## Abbreviations

MG: myasthenia gravis
TOMA: thymoma without myasthenia gravis
TAMG: thymoma-associated myasthenia gravis
TFH: thymic follicular hyperplasia
