# Supplementary material for: Thymic gene expression analysis reveals a potential link between HIF-1A and Th17/Treg imbalance in thymoma associated myasthenia gravis
Source: J Neuroinflammation. 2024 May 11;21:126. doi: 10.1186/s12974-024-03095-7 (PMC11088784; doi:10.1186/s12974-024-03095-7)
Supplement: Supplementary file 1 — Supplementary Material 1 [file 12974_2024_3095_MOESM1_ESM.docx]

**Supplementary tables and figures:**

**Supplementary table 1:**

Primers used in qPCR

| **Gene** | **Forward Primer** | **Reverse Primer** |  |
| --- | --- | --- | --- |
| ***HPRT1*** | GAAAAGGACCCCACGAAGTGT | AGTCAAGGGCATATCCTACAACA |  |
| ***18S*** | GCTTAATTTGACTCAACACGGGA | AGCTATCAATCTGTCAATCCTGTC |  |
| ***RORC*** | GTGGGGACAAGTCGTCTGG | AGTGCTGGCATCGGTTTCG |  |
| ***CCR6*** | CCTGACTTGCATTAGCATGGA | GCGGTAGTGTTCTGGATCGG |  |
| ***IL2*** | AGAACTCAAACCTCTGGAGGAAG | GCTGTCTCATCAGCATATTCACAC |  |
| ***FOXP3*** | AACAGCACATTCCCAGAGTTCCT | CATTGAGTGTCCGCTGCTTCT |  |
| ***HIF1A*** | TATGAGCCAGAAGAACTTTTAGGC | CACCTCTTTTGGCAAGCATCCTG |  |
| ***STAT3*** | \| GATCCAGTCCGTGGAACCAT \| \| --- \| | ATAGCCCATGATGATTTCAGCAA |  |
| ***EP300*** | GGCCTAAACTCTCATCTC | TCTGGTAAGTCGTGCTCCAA |  |
| ***EGLN3*** | CTGGGCAAATACTACGTCAAGG | GACCATCACCGTTGGGGTT |  |
| ***GITR*** | CAGTCCCAGGGGAAATTCAGT | GAACACAGTGAGAAACCCGAA |  |
| ***CTLA4*** | ACGGGACTCTACATCTGCAAGG | GGAGGAAGTCAGAATCTGGGCA |  |

**Supplementary table 2:**

Differently expressed genes between sequenced samples from TAMG and TFH-MG groups.

|  | **log2** | **Std. Err. Log2** | **q Value** |  |
| --- | --- | --- | --- | --- |
|  | **(Fold Change)** | **(Fold Change)** |  |  |
| TBC1D3D | 25.45 | 2.98 | 0.000 | TBC1D3D (TBC1 domain family member 3D) |
| TBC1D3I | 25.45 | 2.98 | 0.000 | TBC1D3I (TBC1 domain family member 3I) |
| TBC1D3K | 25.45 | 2.98 | 0.000 | TBC1D3K (TBC1 domain family member 3K) |
| SNORA20 | 5.88 | 1.5 | 0.032 | SNORA20 (small nucleolar RNA, H/ACA box 20) |
| SNORA71D | 5.84 | 1.51 | 0.036 | SNORA71D (small nucleolar RNA, H/ACA box 71D) |
| SNORD15A | 4.47 | 1.18 | 0.045 | SNORD15A (small nucleolar RNA, C/D box 15A) |
| SNORA11 | 3.97 | 0.85 | 0.003 | SNORA11 (small nucleolar RNA, H/ACA box 11) |
| SNORA8 | 3.65 | 0.97 | 0.046 | SNORA8 (small nucleolar RNA, H/ACA box 8) |
| LOC728989 | 3.62 | 0.81 | 0.006 | LOC728989 (phosphodiesterase 4D interacting protein pseudogene) |
| HSD17B3 | 3.51 | 0.87 | 0.024 | HSD17B3 (hydroxysteroid 17-beta dehydrogenase 3) |
| SNORD17 | 3.46 | 0.85 | 0.023 | SNORD17 (small nucleolar RNA, C/D box 17) |
| FCRL2 | 3.35 | 0.81 | 0.020 | FCRL2 (Fc receptor like 2) |
| FAM153A | 2.77 | 0.68 | 0.022 | FAM153A (family with sequence similarity 153 member A) |
| OSBPL10 | 2.27 | 0.61 | 0.049 | OSBPL10 (oxysterol binding protein like 10) |
| POU6F1 | 2.15 | 0.51 | 0.015 | POU6F1 (POU class 6 homeobox 1) |
| FLT4 | 1.86 | 0.38 | 0.001 | FLT4 (fms related receptor tyrosine kinase 4) |
| MRNIP | **1.75** | 0.34 | 0.000 | MRNIP (MRN complex interacting protein) |
| PRKCE | 1.43 | 0.36 | 0.029 | PRKCE (protein kinase C epsilon) |
| ZMAT1 | 1.38 | 0.35 | 0.029 | ZMAT1 (zinc finger matrin-type 1) |
| LRRK2 | 1.36 | 0.36 | 0.044 | LRRK2 (leucine rich repeat kinase 2) |
| PPFIBP2 | 1.27 | 0.31 | 0.024 | PPFIBP2 (PPFIA binding protein 2) |
| LOC389765 | 1.26 | 0.28 | 0.005 | LOC389765 (kinesin family member 27 pseudogene) |
| PPIP5K1 | 1.2 | 0.3 | 0.022 | PPIP5K1 (diphosphoinositol pentakisphosphate kinase 1) |
| ACCS | 1.19 | 0.32 | 0.049 | ACCS (1-aminocyclopropane-1-carboxylate synthase homolog) |
|  |  |  |  |  |
| GATA3 | -2.32 | 0.53 | 0.010 | GATA3 (GATA binding protein 3) |
| PLK1 | -2.21 | 0.46 | 0.001 | PLK1 (polo like kinase 1) |
| SLC7A5 | -1.95 | 0.51 | 0.044 | SLC7A5 (solute carrier family 7 member 5) |
| BORCS8 | -1.82 | 0.45 | 0.024 | BORCS8 (BLOC-1 related complex subunit 8) |
| SLC35E3 | -1.74 | 0.39 | 0.006 | SLC35E3 (solute carrier family 35 member E3) |
| SSBP3 | -1.57 | 0.41 | 0.037 | SSBP3 (single stranded DNA binding protein 3) |
| JPT1 | -1.5 | 0.36 | 0.020 | JPT1 (Jupiter microtubule associated homolog 1) |
| POLR2A | -1.39 | 0.34 | 0.020 | POLR2A (RNA polymerase II subunit A) |
| PHGDH | -1.32 | 0.26 | 0.000 | PHGDH (phosphoglycerate dehydrogenase) |
| CDK2AP2 | -1.3 | 0.24 | 0.000 | CDK2AP2 (cyclin dependent kinase 2 associated protein 2) |
| BCL2L1 | -1.27 | 0.34 | 0.049 | BCL2L1 (BCL2 like 1) |
| MAZ | -1.22 | 0.3 | 0.024 | MAZ (MYC associated zinc finger protein) |
| SDE2 | -1.22 | 0.23 | 0.000 | SDE2 (SDE2 telomere maintenance homolog) |
| PROSER1 | -1.05 | 0.22 | 0.001 | PROSER1 (proline and serine rich 1) |
| FUS | -0.97 | 0.19 | 0.001 | FUS (FUS RNA binding protein) |
| ZMIZ1 | -0.96 | 0.26 | 0.046 | ZMIZ1 (zinc finger MIZ-type containing 1) |
| ASF1A | -0.91 | 0.23 | 0.032 | ASF1A (anti-silencing function 1A histone chaperone) |
| RAVER1 | -0.9 | 0.14 | 0.000 | RAVER1 (ribonucleoprotein, PTB binding 1) |
| ARF6 | -0.79 | 0.16 | 0.001 | ARF6 (ADP ribosylation factor 6) |
| CNOT11 | -0.75 | 0.2 | 0.044 | CNOT11 (CCR4-NOT transcription complex subunit 11) |
| ARPC5 | -0.7 | 0.16 | 0.005 | ARPC5 (actin related protein 2/3 complex subunit 5) |
| PNRC2 | -0.65 | 0.16 | 0.025 | PNRC2 (proline rich nuclear receptor coactivator 2) |
| VPS26B | -0.63 | 0.13 | 0.001 | VPS26B (VPS26, retromer complex component B) |
| DENND5A | 0.61 | 0.16 | 0.033 | DENND5A (DENN domain containing 5A) |
| C9orf72 | 0.65 | 0.16 | 0.021 | C9orf72 (C9orf72-SMCR8 complex subunit) |
| KRCC1 | 0.65 | 0.16 | 0.029 | KRCC1 (lysine rich coiled-coil 1) |
| FARP2 | 0.76 | 0.19 | 0.020 | FARP2 (FERM, ARH/RhoGEF and pleckstrin domain protein 2) |

**Supplementary figure 1**

**Supplementary figure 2**

**
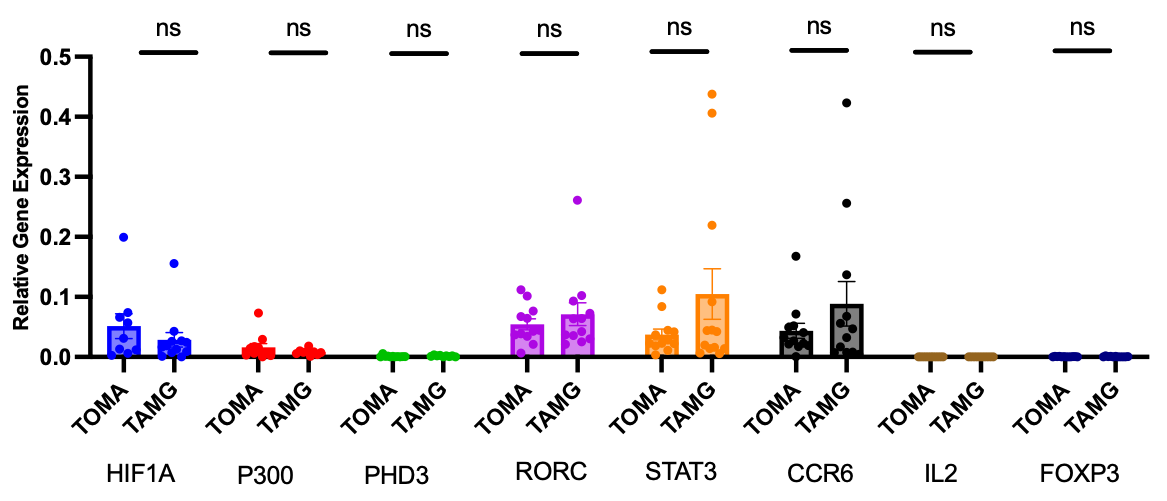
**
